# Menopausal Suicidality and Fragmented Care: A Qualitative Study of Carer and Clinician Perspectives

**DOI:** 10.1111/hex.70840

**Published:** 2026-08-21

**Authors:** Olivia Hendriks, Jason C. McIntyre, Abigail K. Rose, Clair Crockett, Louise Newson, Pooja Saini

**Affiliations:** ^1^ Newson Global Stratford‐Upon‐Avon Warwickshire UK; ^2^ School of Psychology Liverpool John Moores University Liverpool Merseyside UK; ^3^ Newson Clinic Stratford‐Upon‐Avon Warwickshire UK

**Keywords:** caregiving, health services, integrated care, menopause, mental health, qualitative research, suicidality

## Abstract

**Background:**

Emerging evidence indicates elevated suicidality during the menopausal transition, yet little is known about its impact on those providing support.

**Objective:**

To explore how menopausal suicidality is experienced by informal carers and menopause‐focused healthcare professionals, and to identify relational and service‐level consequences.

**Design:**

Qualitative study using semi‐structured interviews and reflexive thematic analysis.

**Setting and Participants:**

Twenty‐eight participants were recruited: 19 healthcare professionals working within a specialist menopause clinic and 9 informal carers supporting women who had experienced suicidality during menopause. Interviews were conducted online.

**Main Variables Studied:**

Participants' accounts of menopausal suicidality, caregiving burden, clinical responsibility and experiences of healthcare systems.

**Results:**

Five interrelated themes were identified. Menopausal suicidality was described as cyclical, hidden and sometimes acute, with hormonally patterned fluctuations and perceived impulsivity. Emotional labour shifted into families, generating vigilance, fear and exhaustion. Clinicians described managing suicide risk within services not designed for crisis care. Participants also reported fragmented pathways in which women fell between hormonal and psychiatric services, and called for more integrated, anticipatory support.

**Discussion and Conclusions:**

Menopausal suicidality exposes gaps between endocrine and mental healthcare, diffusing responsibility across services and increasing burden on carers and clinicians. Integrated care pathways, menopause‐specific suicide prevention training and support for informal and professional caregivers are needed. To our knowledge, this is the first qualitative study to examine caregiving experiences in menopausal suicidality.

**Patient or Public Contribution:**

A Public Advisory Group with lived and professional experience informed the study focus and prioritised exploration of carer and clinician perspectives.

## 1 Introduction

Menopause is a significant transitional phase in a woman's life, marked by hormonal changes that can produce a wide range of physical and psychological symptoms [1]. Although physical symptoms such as hot flushes and sleep disturbance are widely recognised, the mental health implications of menopause have only recently received sustained clinical and research attention [2]. Women may be at increased risk of depression during perimenopause and menopause [3], while population statistics indicate that women's age‐specific suicide rates peak during midlife, broadly coinciding with the menopausal transition [4].

Emerging evidence has begun to clarify both the scale and potentially distinct nature of psychological distress and suicide risk during this reproductive stage. Systematic reviews indicate that depressive symptoms and suicidality may increase during the menopausal transition compared with premenopause [5, 6]. International clinical guidelines also recognise perimenopausal depression as a presentation that may require tailored assessment and management because hormonal fluctuations can influence the onset, severity, and expression of affective symptoms [2]. Declining and fluctuating oestrogen and progesterone are thought to influence serotonergic, dopaminergic, and stress‐response systems involved in mood regulation [7, 8]. For some women, these neuroendocrine changes may contribute to the emergence or worsening of affective symptoms, including among those without a prior psychiatric history [2, 9].

Despite increasing recognition of menopause‐related mental health difficulties, relevant care pathways remain poorly integrated. Women experiencing severe psychological symptoms may be treated within mental health services where hormonal contributors are not routinely considered, while menopause services are often not configured to assess and manage acute psychiatric risk [10, 11]. Management may include hormone replacement therapy (HRT), psychological therapies, antidepressant medication where appropriate, and lifestyle interventions [12]. Evidence suggests that HRT may improve depressive symptoms in some perimenopausal women, particularly when symptoms are temporally associated with the menopausal transition [13, 14]. Nevertheless, menopause‐related psychological symptoms may be overlooked or attributed primarily to psychiatric or psychosocial causes, potentially delaying recognition of hormonal contributors and access to appropriate treatment [9]. In a recent cohort of 957 women attending a specialist menopause service, approximately one in six reported suicidal ideation at their first appointment [15].

Research on menopausal suicidality has predominantly focused on women's individual experiences, with less attention paid to its relational and systemic consequences. Serious mental health difficulties can affect family functioning, caregiving roles, healthcare engagement, and treatment outcomes [16, 17]. Informal carers supporting people experiencing suicidality frequently report substantial psychological strain, including guilt, shame, powerlessness, depression, and, in some cases, suicidal ideation themselves [18, 19, 20, 21]. They may also play a central role in recognising deterioration, facilitating help‐seeking, advocating for the person at risk, and navigating fragmented healthcare systems [16, 19, 22].

Healthcare professionals may face related challenges. Clinicians in primary care often report low confidence in suicide risk assessment and management, citing limited training and perceived lack of competence in responding to suicidal presentations [23, 24, 25]. These difficulties may be particularly salient in menopause care, where clinicians can encounter severe psychological distress that does not fit neatly within traditional psychiatric frameworks while working in services not designed to manage acute suicide risk. This may create a dual burden of clinical uncertainty and organisational unpreparedness.

Although caregiver burden has been examined in severe mental illness and suicidality [26, 27], this literature rarely considers the hormonal, midlife, and gendered dimensions of menopausal distress. Suicidality during menopause may emerge without prior psychiatric history, fluctuate in association with hormonal changes, and disrupt established family roles and expectations [6]. It can also unfold across fragmented hormonal and mental health services, leaving families and clinicians to respond without clear frameworks or coordinated support [28]. Little is therefore known about how informal carers and menopause‐focused clinicians experience and respond to suicidality during the menopausal transition, or how fragmented care shapes their roles, wellbeing, and support needs.

Understanding these perspectives is important for developing patient‐ and family‐centred models of care that incorporate the experiences of patients and those closest to them into service design and delivery [29, 30]. Examining carers' and clinicians' experiences may help identify how menopause services and suicide prevention pathways could be better integrated to support women and those around them [31, 32]. Discussions within the study's advisory group further reinforced the importance of examining perspectives that remain largely absent from menopause and suicidality research.

This study, therefore, explores the experiences of informal carers and menopause‐focused clinicians supporting women experiencing suicidality during the menopausal transition. By examining personal and professional caregiving perspectives, it aims to illuminate the emotional labour, systemic tensions, and unmet support needs that shape care for women experiencing menopause‐related suicidality.

## 2 Method

### 2.1 Design

This study employed a qualitative design using semi‐structured interviews to explore the experiences and perspectives of informal carers and menopause‐focused clinicians supporting women reporting suicidality during the menopausal transition. An interpretive approach was adopted to enable in‐depth exploration of relational, emotional, and systemic dimensions of caregiving within this context. Semi‐structured interviews were selected to allow participants to describe their experiences in their own terms while providing sufficient flexibility to discuss sensitive and complex issues related to suicidality.

Interviews were conducted between March 2023 and February 2024 to maximise accessibility and geographical reach.

### 2.2 Participants

Twenty‐eight participants were interviewed: 19 menopause healthcare professionals (MHCPs) working within a specialist private menopause clinic and nine informal carers supporting women who had experienced suicidality during menopause. The MHCP group comprised 10 general practitioners, five administrative staff, three pharmacists and one nurse. Administrative staff were included because they formed part of the patient care pathway, frequently responding to women experiencing severe psychological distress, facilitating clinician access and coordinating urgent appointments. The informal carer group comprised six husbands and three friends, recruited through the clinic and public advertisements. Participant roles and relationships are summarised in Table 1.

**Table 1:** Participant roles and relationships.

Participant group	Role/relationship	Number (*n*)
Menopause Healthcare Professionals (MHCPs)	General practitioners	10
	Administrative staff	5
	Pharmacists	3
	Nurse	1
Subtotal MHCPs		19
Informal carers	Husbands	6
	Friends	3
Subtotal carers		9
Total participants		28

Sampling sought variation across professional roles and personal relationships, with all participants having direct experience of supporting women with menopause‐related psychological distress. Eighteen MHCPs were women, and one was a male pharmacist. To reduce the risk of deductive disclosure within a small specialist community, detailed demographic data, including age, ethnicity and location, were not systematically collected. Professional roles and relationships were retained to provide contextual information while protecting confidentiality. Quotations are labelled IC for informal carers and MHCP for menopause healthcare professionals.

### 2.3 Materials

A semi‐structured interview schedule was developed to explore participants' experiences of supporting women experiencing suicidality during the menopausal transition. The guide was informed by existing literature on menopausal mental health and suicidality, as well as findings from related research within this programme of work. Open‐ended questions were designed to elicit detailed accounts while allowing flexibility to follow participant‐led narratives.

Key domains included: (1) experiences of supporting women during menopause, particularly in the context of severe psychological distress; (2) challenges encountered in managing or responding to suicidality; (3) interactions with healthcare systems and perceptions of available support; (4) strategies or approaches used during mental health crises; and (5) reflections on gaps and potential improvements within menopause care pathways.

Questions were adapted as appropriate for professional and informal carers to ensure relevance to participants' roles and lived contexts.

### 2.4 Procedure

Menopause healthcare professionals (MHCPs) were recruited through a specialist private menopause clinic via an internal study invitation. Interested staff contacted the lead researcher directly, and participation was voluntary and independent of their employment. Informal carers were recruited through information shared with eligible individuals by MHCPs and targeted social media advertisements. Potential participants contacted the researcher directly, ensuring that clinic staff were unaware of who chose to participate.

Participants received an information sheet outlining the study aims, procedures, confidentiality, withdrawal rights and available support. Written consent and audio‐recorded verbal confirmation of consent were obtained before each interview. Participants were reminded that they could decline questions, pause or end the interview without consequence.

Individual interviews were conducted via Microsoft Teams by the first author and lasted approximately 30–45 min. The first author is an experienced psychological researcher with clinical training and experience in suicide risk assessment and supporting individuals experiencing acute psychological distress. Participant wellbeing was monitored throughout, with plans to pause or terminate interviews if significant distress arose. Following each interview, participants were debriefed and provided with information about relevant support services. No interviews were terminated because of distress, and no safeguarding concerns arose.

### 2.5 Patient and Public Involvement (PPI)

PPI informed the development of this research programme throughout the PhD project. Two advisory groups were established: a Public Advisory Group comprising 14 members with lived experience of menopausal mental health challenges alongside professionals working in suicide prevention and menopause care, and a professional steering group of eight clinicians and researchers. These groups met periodically throughout the research programme to advise on research priorities, study design, interpretation of findings, and dissemination.

For the present study, feedback from the advisory groups informed the decision to explore the experiences of both informal carers and menopause healthcare professionals, reflecting the importance of understanding the wider relational and service context surrounding menopausal suicidality. Advisory group discussions also informed refinement of the semi‐structured interview schedule, including the exploration of service fragmentation, barriers to accessing care, and the experiences of supporting women experiencing severe psychological distress. Following data analysis, emerging themes were discussed with the advisory groups to support interpretation of the findings and ensure that the analysis reflected issues considered meaningful and relevant by those with lived and professional experience.

### 2.6 Data Analysis Strategy

Interviews were transcribed verbatim and analysed using reflexive thematic analysis following Braun and Clarke [33]. Analysis was primarily inductive and interpretive, with attention to both semantic and latent meanings.

The first author developed familiarity with the data through repeated reading and note‐taking. Menopause healthcare professional and informal carer transcripts were initially coded separately to preserve contextual differences, before patterns were compared across groups to identify convergence, divergence and shared relational or systemic meanings. Candidate themes were developed iteratively through recursive engagement with the transcripts, with overlapping themes combined and thematic boundaries and definitions refined to ensure coherence and distinction.

NVivo 14 [34] supported data organisation and retrieval. Analytic discussions with the wider research team encouraged reflexive scrutiny of developing interpretations rather than consensus‐based validation, and key analytic decisions were documented throughout. Themes were ultimately defined and named to capture patterned meanings across and between participant groups.

### 2.7 Researcher Positioning

The first author has a professional background in suicide prevention and psychological intervention, alongside academic expertise in menopause‐related mental health and suicidality. This clinical experience informed sensitivity to discussions of risk, safeguarding, and service navigation during interviews. Reflexive attention was paid to how prior training in suicide risk assessment and crisis management might shape both questioning and interpretation. Analytic dialogue within the research team supported critical examination of assumptions and alternative readings of participants' accounts.

### 2.8 Ethical Considerations

This study was conducted in accordance with the Declaration of Helsinki (1975, revised 2013) and received ethical approval from Liverpool John Moores University Research Ethics Committee (LJMU REC ID: 22/PSY/080). All participants received a detailed information sheet outlining the study aims, confidentiality procedures, and their right to withdraw at any time without consequence. Written and audio‐recorded informed consent was obtained prior to interview. Given the sensitive focus on suicidality, interviews were conducted with particular attention to participant wellbeing. Participants were reminded they could pause or discontinue the interview at any point, and support resources were provided following participation. In the event of distress or disclosure of ongoing risk, a pre‐defined safeguarding protocol was in place. All data were anonymised at transcription and stored securely in accordance with institutional data protection guidelines.

## 3 Findings

Five interrelated themes illuminate how menopausal suicidality reverberates across relational and clinical contexts. These themes reflect: (1) the cyclical, hidden, and acute nature of suicidality during menopause; (2) the redistribution of emotional and moral labour within families and (3) clinical roles; (4) fragmented systems in which women and carers fall between hormonal and psychiatric care; and (5) carers' reflections on what more integrated and responsive support might look like.

## 4 Menopausal Suicidality as Cyclical, Hidden, and Acute

Caregivers described menopausal suicidality as both prevalent and severe, encompassing self‐harm, persistent ideation, and suicide attempts. Often, these struggles only came to light retrospectively, as many women had previously hesitated to share their experiences or lacked the opportunity to do so in healthcare settings or with loved ones. As one participant observed, ‘more people have got the language to put to it now’ (IC 4), reflecting a changing environment in recognising and articulating these challenges. These accounts suggest that menopausal suicidality was often experienced as partially concealed or misrecognised, with language and understanding emerging only in hindsight. This delayed recognition contributed to uncertainty among carers, who frequently felt they were responding to crises without prior warning or shared conceptual frameworks.

A clear distinction was noted between passive suicidal thoughts, such as ‘Why am I here? What's the point of it all?’ (IC 2), and active suicidal ideation involving specific plans to end one's life. Clinicians suggested that those with active plans often refrain from seeking help, as they ‘just want to go and do it’ (MHCP 12), leaving MHCPs primarily supporting individuals described as ‘desperate’ (MHCP 27) for support rather than actively intent on dying. The distinction between passive and active ideation shaped how risk was perceived and managed. While passive thoughts were often framed as expressions of despair or overwhelm, active intent was described as more private and less likely to be disclosed, complicating opportunities for timely intervention.

Crucially, participants described this distress as temporally patterned, with predictable exacerbations in the premenstrual phase. As one husband explained, ‘it was always in the one or two weeks before her period’ (IC 8), illustrating the perceived regularity of these fluctuations. Suicidality was therefore experienced not as a static depressive state, but as hormonally fluctuating and episodic. This cyclical quality created both urgency and ambiguity for carers, who attempted to anticipate periods of heightened risk without structured guidance or integrated clinical pathways, highlighting the need for proactive planning and targeted support during these periods of heightened vulnerability.

The impulsive nature of suicidality was another significant concern, with women sometimes acting without warning. Five caregivers reported repeated suicide attempts among the women they supported, and collectively reported seven attempts that resulted in death. The coexistence of cyclical patterning and perceived impulsivity generated a paradox for carers: while some risk periods appeared predictable, attempts could still occur abruptly, reinforcing a sense of fragility and limited control.

## 5 Redistributed Emotional Labour Within the Family

Caring for menopausal women experiencing suicidality placed a sustained emotional, psychological, and practical burden on loved ones, reshaping their daily routines and sense of stability. As one participant reflected, ‘It's not something that happens to you, and yet it is something that happens to you because you live with it every day’ (IC 3). This captures how suicidality, although not directly experienced by the caregiver, became an ever‐present feature of family life.

Participants described caregiving as emotionally destabilising and chronically exhausting, characterised by vigilance, anticipatory fear, and persistent self‐doubt. Many worried constantly about their loved one's safety, feeling ‘scared of not being able to find the solution’ (IC 7) or ‘not knowing what to do’ (IC 1). For some, exposure to suicidal statements or episodes of heightened distress was itself triggering, particularly among those with prior experiences of mental health difficulties or abuse. Over time, this generated a state of ongoing psychological surveillance and anxiety, as carers monitored mood shifts and behaviour in an attempt to prevent escalation, often at the expense of their own wellbeing.

Balancing this vigilance alongside employment, childcare, and other family responsibilities intensified the strain. Several participants described feeling ‘stretched thin’ (IC 7), navigating their roles with limited support and sometimes taking time off work to manage crises. The cumulative effect was a profound sense of emotional depletion and inadequacy, with carers questioning whether they had done enough or could have acted differently.

These accounts suggest that menopausal suicidality redistributed sustained emotional labour within the household, transforming intimate relationships into sites of risk management and crisis containment.

## 6 Clinicians at the Edge of Crisis Care

Menopause healthcare professionals (MHCPs) described supporting suicidal menopausal women as emotionally demanding and professionally complex. While their roles differed from those of family members, they reported comparable strain, often feeling ‘vulnerable’, ‘anxious’, or ‘drained’ (MHCP 19), effects that extended beyond working hours. Maintaining professional boundaries proved challenging, particularly when cases involved repeated crises or perceived high risk. The confidentiality of their role further limited opportunities to process these experiences with others, intensifying feelings of isolation.

Clinicians frequently described feeling ‘out of their depth’ (MHCP 22) when navigating suicidality within a service not designed as a crisis provider. The geographical dispersion of patients complicated referrals to appropriate local services, leaving clinicians uncertain about the adequacy of their interventions. Some recalled particularly ‘dreadful’ (MHCP 26) cases that remained with them long after consultations ended, suggesting the enduring emotional imprint of this work.

Workplace structures compounded these pressures as clinicians described ‘working a lot of overtime’ (MHCP 18) to manage vulnerable patients and responding to acute distress despite operating outside formal emergency pathways. Remote consultations added further complexity, with participants noting the difficulty of assessing risk via phone or video. Administrative staff, often the first point of contact, encountered highly distressed patients without clinical training, raising concerns about their exposure to risk and emotional burden. As one MHCP reflected, ‘The hardest part is… when do you feel that you can hang up the phone?’ (MHCP 26). This question captures the moral tension inherent in providing care at the margins of crisis services, balancing professional limits against perceived responsibility for patient safety.

These accounts position menopause clinicians at the edge of crisis care: encountering acute suicidality within a service model not structurally equipped to contain it.

## 7 Fragmented Systems: Falling Between Hormonal and Psychiatric Care

A majority of participants described navigating a healthcare system in which the connection between menopause and mental health was frequently overlooked. Participants reported that hormonal factors were rarely considered within mental health assessments, with suicidality often attributed solely to depression. Symptoms such as menstrual irregularity or heavy bleeding were described as dismissed, and blood tests – despite their limitations during perimenopause – were sometimes used to rule menopause out entirely. This left carers feeling invalidated and unheard, describing encounters as ‘hostile’ and ‘painful’, (IC 4) with some reporting being ‘talked down to’ (IC 3) or repeatedly ‘fobbed off’ (IC 9).

One participant recalled their loved one's experience: ‘They just sedated her, and sedated her, more, and more, and more’ (IC 6), reflecting what was perceived as an overreliance on psychiatric medication without exploration of hormonal contributors. Another carer described a fundamental disconnect: ‘There was not a single conversation between what was happening medically and mentally’ (IC 2). Participants emphasised a lack of communication across gynaecology, psychiatry, psychology, and endocrinology, leaving women to navigate fragmented pathways with no clear point of ownership.

Participants characterised being caught between services, where distress was not considered acute enough for crisis intervention, yet remained inadequately addressed in routine care. As one MHCP noted, ‘Frankly, you need to attempt to harm yourself to get admitted’ (MHCP 28). This positioning created a perceived threshold effect, in which intervention appeared contingent upon escalation rather than prevention.

Access to hormone replacement therapy (HRT) further illustrated systemic disjunction. Several carers reported that hormonal treatment was considered only after multiple psychiatric medications, and in some cases, electroconvulsive therapy, had failed. Shortages, limited confidence, and reluctance among some GPs to prescribe HRT compounded these difficulties. One MHCP described patients concealing their HRT use from their GP out of fear of damaging the therapeutic relationship, highlighting fragile integration between services. In another account, a participant—himself a GP—described personally sourcing HRT for his wife and bringing it to the hospital so it could be entered into the prescribing system, as clinicians were uncertain how to obtain or manage it. Such accounts emphasise the extent to which responsibility shifted onto families when institutional pathways faltered.

Across accounts, participants attributed these challenges to limited training in menopause and its psychological dimensions. Many clinicians reflected that menopause had received minimal coverage in their education, particularly regarding its interaction with suicidality. As one clinician questioned, ‘It's something that affects every single woman in the world, so why are we not teaching it?’ (MHCP 20).

Collectively, these narratives depict a system in which responsibility for hormonally mediated psychological distress is dispersed rather than integrated. Suicidal menopausal women were perceived as falling between hormonal and psychiatric care, with neither domain fully equipped—or mandated—to hold the complexity of their presentations.

## 8 Imagining Integrated Futures

Across both informal and clinical accounts, participants articulated a desire for more integrated, anticipatory forms of support. Rather than responding only at points of crisis, carers emphasised the need for practical tools to navigate conversations about menopause and mental health within the family context. Several described feeling unsure ‘where to start’, (IC 4) particularly when explaining suicidality to children or other family members. Participants suggested that services should move beyond an exclusively individualised model of treatment to one that recognises and actively involves partners and families in care planning and psychoeducation. Practical supports, such as temporary childcare or respite provision, were viewed as essential in alleviating the intensity of sustained caregiving.

Participants also highlighted the value of peer connection, proposing dedicated communities for spouses and family members to share strategies and reduce isolation. At a structural level, many argued that ‘more work needs to be done between psychiatrists, psychologists, and menopause specialists’ (MHCP 11), reflecting a desire to bridge the divide between hormonal and psychiatric care identified in earlier themes. Suggestions included embedding psychological expertise within menopause services or establishing specialist pathways explicitly addressing hormonally mediated mental distress.

Financial barriers were also raised, with MHCPs proposing mechanisms such as hardship funds or ‘pay it forward’ (MHCP 27) schemes to improve equitable access to private menopause care. More broadly, participants called for comprehensive education for healthcare professionals on the interaction between hormonal and mental health. As one carer observed, ‘The menopause, it will happen to 50% of the country, and then the rest of the country have to deal with it’ (MHCP 16), underscoring the perceived societal scale of the issue.

Collectively, these proposals reflect an attempt to reimagine menopause care as integrated, preventative, and relational, recognising not only the woman experiencing distress but the wider family and service structures through which support is delivered.

## 9 Discussion

Our study explored how suicidality during the menopausal transition reverberates beyond the individual, reshaping the emotional, relational, and professional worlds of those providing support. To our knowledge, this is the first qualitative study to examine how menopausal suicidality affects both informal carers and menopause‐focused clinicians, highlighting the relational and systemic consequences of hormonally mediated psychological distress. Consistent with wider caregiving literature, participants described distress, exhaustion, fear, and guilt; experiences also documented among carers in contexts such as cancer and dementia [35, 36, 37]. However, the present findings extend this literature by highlighting a distinct configuration of caregiver burden shaped by hormonally fluctuating distress and fragmented service pathways. Unlike more stable chronic conditions, menopausal suicidality was described as cyclical, temporally patterned, and at times impulsive, generating sustained vigilance within families and professional uncertainty among clinicians.

Participants consistently described the absence of integrated hormonal and psychiatric care as a defining feature of their experiences, leaving carers feeling unsupported and responsible for navigating disconnected services. While previous research has documented emotional burden and elevated suicide risk among carers supporting individuals who self‐harm [38, 39, 40], these studies have largely conceptualised caregiver burden as a consequence of managing psychiatric illness. Our findings suggest that menopausal suicidality introduces an additional layer of complexity because responsibility is often dispersed across endocrine and mental health services, creating uncertainty regarding where care should be sought and who should assume clinical responsibility. This interpretation aligns with broader health services literature demonstrating that fragmented healthcare systems often shift responsibility for coordinating care onto patients and families, whereas integrated, person‐centred models improve continuity, coordination, and patient experiences across services [41, 42]. Taken together, these findings suggest that fragmentation itself may act as a mechanism through which caregiver burden is intensified during menopausal suicidality. Our findings, therefore, extend existing caregiving literature by identifying health system fragmentation—not simply the emotional demands of caregiving—as an important contributor to caregiver burden during menopausal suicidality.

Caregivers also described significant financial strain, particularly where job loss or reduced working hours coincided with the need to pursue private menopause care. Although the economic burden of caregiving is well documented across chronic illness contexts [43], our findings suggest that financial strain associated with menopausal suicidality may arise through different mechanisms. Rather than reflecting the costs of long‐term caregiving alone, participants described financial hardship resulting from structural barriers to accessing timely menopause care, including prolonged National Health Service (NHS) waiting times, hormone replacement therapy (HRT) shortages, and the need to self‐fund specialist consultations [44, 45]. This suggests that existing inequities in menopause care may compound the emotional and practical burden experienced by families, disproportionately affecting those without the financial resources to access private treatment. Consequently, our findings extend the caregiving literature by highlighting healthcare accessibility and affordability as important contributors to caregiver burden during menopausal suicidality.

Previous research has shown that stigma surrounding suicide within the family can create an atmosphere of silence, shame, and fear, limiting opportunities for open discussion and support [22, 46]. Our findings reinforce the importance of these processes but suggest that stigma may be particularly complex in the context of menopausal suicidality, where symptoms are often poorly understood and frequently misattributed to personality, ageing, or psychosocial stressors rather than recognised as potentially hormone‐related [47]. Participants described uncertainty about how to discuss suicidality with children and other family members, reflecting both the stigma surrounding suicide itself and the broader lack of public understanding of menopause‐related psychological distress. These findings extend previous literature by highlighting how suicide stigma may intersect with menopause stigma, creating additional barriers to help‐seeking and family communication. Services should therefore consider addressing both forms of stigma through family‐inclusive education and practical guidance that equips caregivers to have open, age‐appropriate conversations about suicidality within the home.

Similar emotional challenges have been reported among healthcare professionals working in crisis and trauma settings, where repeated exposure to suicide risk is associated with compassion fatigue, burnout, and feelings of helplessness [48, 49]. Our findings suggest that menopause healthcare professionals experience many of these same emotional demands but within a markedly different clinical context. Unlike mental health practitioners, menopause clinicians are primarily trained to manage hormonal health rather than acute psychiatric crises, yet participants described increasingly finding themselves responsible for assessing suicide risk and supporting women in severe psychological distress. This role expansion appears to reflect wider systemic gaps in the recognition and management of hormonally mediated mental health difficulties, alongside limited education on the psychological effects of menopause across healthcare [8, 50]. Although healthcare professionals outside mental health services should not be expected to manage suicidal crises independently [51], our findings suggest that menopause clinicians are increasingly required to do so because women experiencing hormonally related suicidality often fall between endocrine and psychiatric services. This highlights a previously under‐recognised ‘double burden’, whereby clinicians must simultaneously develop expertise in menopause management while responding to suicide risk within systems that provide limited training or support in either domain. These findings therefore extend existing literature on clinician burnout by identifying role ambiguity and fragmented care pathways as additional contributors to professional strain, emphasising the need for menopause‐specific suicide prevention training and clearer multidisciplinary care pathways.

Participants consistently described a siloed system in which hormonal and psychiatric care operated in parallel rather than in partnership. While multidisciplinary models have been shown to improve diagnostic clarity, continuity of care, and patient outcomes across a range of clinical settings, including for individuals experiencing suicidality [32, 52], our findings suggest that these principles have yet to be fully realised in menopause care. Rather than functioning as complementary services, participants described endocrine and mental health pathways as operating independently, leaving uncertainty regarding clinical responsibility and increasing the burden placed on women, carers, and menopause clinicians to coordinate care across disconnected systems. These findings extend previous literature on integrated care by suggesting that the consequences of health system fragmentation may be particularly pronounced when suicidality occurs alongside hormonally mediated psychological distress. In these situations, effective care requires coordination across menopause and mental health services, yet participants consistently described these pathways as operating in isolation rather than in partnership [10, 11, 42]. In menopause‐related contexts, closer collaboration between primary care, gynaecology, endocrinology, psychiatry, and psychology may therefore improve continuity of care, reduce fragmentation, and alleviate caregiver burden.

The absence of dedicated support structures for carers further contributed to feelings of isolation, with many participants describing a lack of opportunities to share experiences or receive guidance from others facing similar challenges. Evidence from dementia and other chronic illness contexts demonstrates that peer support groups can reduce isolation, enhance coping, and improve caregiver wellbeing [53, 54]. Our findings suggest that similar interventions may also benefit families supporting women experiencing menopausal suicidality; however, unlike other caregiving contexts, such resources are currently scarce and rarely tailored to the unique challenges associated with menopause‐related psychological distress. While initiatives such as the UK Menopause Café, a community‐led peer support initiative that encourages open discussion about menopause and welcomes partners and family members, may provide informal sources of support, there is currently limited empirical evaluation of their effectiveness or their ability to meet the specific needs of carers supporting women experiencing suicidality [55]. These findings, therefore, highlight an important gap in service provision and suggest that developing and evaluating menopause‐specific, carer‐inclusive support programmes should be a priority for future research and service development.

In addition to addressing the support needs of unpaid carers, our findings highlight the importance of providing structured clinical supervision for menopause healthcare professionals, many of whom described feeling overwhelmed by the complexity of supporting women experiencing suicidality. Clinical supervision is well established within mental health services, where it is associated with improved staff wellbeing, reduced burnout, enhanced job satisfaction, and safer management of high‐risk patients [56, 57]. However, such support is less commonly embedded within non‐mental health settings [58]. Our findings suggest that menopause clinicians increasingly occupy a hybrid role, providing specialist hormonal care while also responding to acute psychological crises, yet without the supervision structures typically available to mental health professionals. This extends previous literature by highlighting an important mismatch between the responsibilities placed upon menopause clinicians and the organisational support available to them. Given the elevated prevalence of suicidality during the menopausal transition [6], menopause services may benefit from structured supervision frameworks that enable clinicians to reflect on high‐risk cases, strengthen confidence in managing suicidal distress, and mitigate the emotional impact of repeated exposure to psychologically complex presentations.

Overall, our findings suggest that menopausal suicidality exposes important structural gaps in the integration of hormonal and mental health care. Responsibility for recognising and managing suicide risk appeared to be diffused across services, leaving families and menopause clinicians to absorb the emotional, practical, and clinical consequences of fragmented care. By incorporating the perspectives of both informal carers and menopause healthcare professionals, this study extends existing research beyond women's individual experiences to demonstrate the wider relational and system‐level impact of menopausal suicidality. Addressing these challenges requires not only improved education and training but also integrated models of care that recognise the intersecting endocrine, psychological, and relational dimensions of midlife mental health.

## 10 Limitations

This study has several limitations. The sample included menopause healthcare professionals primarily working within a specialist private clinic, which may limit transferability to public healthcare settings where organisational structures, resource availability, and patient pathways differ. Although social media recruitment broadened participation among informal carers, the experiences captured may not reflect those of carers from more socioeconomically or culturally diverse backgrounds. Also, demographic data were limited in order to protect participant anonymity, which may constrain analysis of how age, ethnicity, or career stage shaped experiences.

Additionally, while this study illuminates relational and systemic impacts of menopausal suicidality, it does not capture the perspectives of women currently experiencing acute suicidal crisis, nor does it examine longitudinal caregiver trajectories over time. Future research across varied healthcare contexts and cultural settings would deepen understanding of how structural inequities shape access to integrated hormonal and mental health care.

## 11 Implications for Clinical Practice and Service Development

Our findings highlight several opportunities to strengthen the identification, management, and prevention of menopausal suicidality through improvements in service organisation, workforce development, and family support.

Embedding mental health professionals within menopause services represents one practical approach to achieving this integration. Collaborative care models, in which mental health professionals work alongside other clinicians to deliver coordinated assessment and management, have consistently been associated with improved outcomes for people experiencing depression and other common mental health conditions in primary care [59].

Beyond collaborative care, system‐wide approaches to suicide prevention emphasise the importance of integrated, multidisciplinary care pathways to improve continuity of care and reduce suicide risk [60]. Adapting these principles to menopause services may facilitate earlier recognition of hormonally mediated psychological distress, improve coordination between menopause and mental health services, and enhance continuity of care for women experiencing suicidality.

Emerging guidance and conceptual models within PMDD emphasise the importance of recognising the interaction between hormonal fluctuations and suicide risk and advocate menstrual cycle‐informed, multidisciplinary approaches to assessment and management [61] (Matthews et al., 2026). Although PMDD and menopause are distinct reproductive conditions, both involve hormonally mediated changes that can influence psychological wellbeing and suicide risk. Consequently, menstrual cycle‐informed, multidisciplinary approaches developed within PMDD may provide a useful framework for informing menopause services.

Structured psychoeducation, peer support programmes, and family‐inclusive interventions have demonstrated benefits in other caregiving contexts by reducing isolation and improving coping [53, 54]. Adapting similar models for families supporting women experiencing menopausal suicidality represents a feasible and evidence‐informed strategy to reduce caregiver burden while improving families' confidence in recognising and responding to psychological crises.

Supporting the menopause workforce is also essential. Menopause healthcare professionals require workplace structures that acknowledge the emotional toll of managing suicidality. Routine reflective supervision, regular multidisciplinary case discussions, and clearly defined escalation pathways are widely used in mental health settings to support clinicians managing suicide risk [56, 57]. Introducing similar structures within specialist menopause services may strengthen clinician confidence and improve patient safety.

Collectively, these recommendations move beyond individual clinician education towards system‐level redesign that better integrates hormonal and mental healthcare. Strengthening service integration, workforce education, structured support for carers, and organisational support for clinicians has the potential to reduce fragmentation, improve continuity of care, and ultimately improve outcomes for women experiencing menopause‐related suicidality.

## Author Contributions

**Olivia Hendriks:** investigation, writing – original draft, methodology, validation, visualisation, writing – review and editing, formal analysis, data curation, software. **Abigail K. Rose:** Supervision, writing – review and editing. **Jason C. McIntyre:** writing – review and editing, supervision. **Clair Crockett:** conceptualisation, resources, project administration. **Louise Newson:** conceptualisation, resources, project administration. **Pooja Saini:** conceptualisation, funding acquisition, writing – review and editing, supervision.

## Ethics Statement

This study was conducted in accordance with the Declaration of Helsinki (1975, revised 2013) and received ethical approval from Liverpool John Moores University Research Ethics Committee (LJMU REC ID: 22/PSY/080). All participants provided informed consent prior to interview. Safeguards were implemented to support participant wellbeing when discussing sensitive topics, including the provision of information about relevant support services.

## Conflicts of Interest

The authors declare no conflicts of interest.

## Data Availability

The data supporting the findings of this study are available within the article and its supporting informations. Additional data may be available from the corresponding author upon reasonable request.
